# Cercosporamide inhibits bone morphogenetic protein receptor type I kinase activity in zebrafish

**DOI:** 10.1242/dmm.045971

**Published:** 2020-09-24

**Authors:** Jelmer Hoeksma, Gerard C. M. van der Zon, Peter ten Dijke, Jeroen den Hertog

**Affiliations:** 1 Hubrecht Institute – KNAW and University Medical Center Utrecht, 3584 CT Utrecht, The Netherlands; 2 Department of Cell and Chemical Biology, Leiden University Medical Center, 2333 ZC Leiden, The Netherlands; 3 Oncode Institute, Leiden University Medical Center, 2333 ZC Leiden, The Netherlands; 4 Institute Biology Leiden, Leiden University, 2333 BE Leiden, The Netherlands

**Keywords:** Bone morphogenetic protein, Cercosporamide, Dorsomorphin, Kinase inhibitor, Zebrafish

## Abstract

Zebrafish models are well-established tools for investigating the underlying mechanisms of diseases. Here, we identified cercosporamide, a metabolite from the fungus *Ascochyta aquiliqiae*, as a potent bone morphogenetic protein receptor (BMPR) type I kinase inhibitor through a zebrafish embryo phenotypic screen. The developmental defects in zebrafish, including lack of the ventral fin, induced by cercosporamide were strikingly similar to the phenotypes caused by renowned small-molecule BMPR type I kinase inhibitors and inactivating mutations in zebrafish BMPRs. In mammalian cell-based assays, cercosporamide blocked BMP/SMAD-dependent transcriptional reporter activity and BMP-induced SMAD1/5-phosphorylation. Biochemical assays with a panel of purified recombinant kinases demonstrated that cercosporamide directly inhibited kinase activity of type I BMPRs [also called activin receptor-like kinases (ALKs)]. In mammalian cells, cercosporamide selectively inhibited constitutively active BMPR type I-induced SMAD1/5 phosphorylation. Importantly, cercosporamide rescued the developmental defects caused by constitutively active Alk2 in zebrafish embryos. We believe that cercosporamide could be the first of a new class of molecules with potential to be developed further for clinical use against diseases that are causally linked to overactivation of BMPR signaling, including fibrodysplasia ossificans progressiva and diffuse intrinsic pontine glioma.

This article has an associated First Person interview with the first author of the paper.

## Abstract (precis)

**Summary:** Cercosporamide, a metabolite from the fungus *Ascochyta aquiliqiae*, was identified as a potent bone morphogenetic protein receptor (BMPR) type I kinase inhibitor through a zebrafish embryo phenotypic screen.

## INTRODUCTION

Zebrafish (*Danio rerio*) is an attractive model for studying the biological effects of genetic mutations and chemical compounds *in vivo*. Zebrafish are vertebrates with a highly conserved physiology that develop all organs and primary tissues in several days (Kimmel et al., 1995). Moreover, zebrafish embryos are transparent, which makes development easy to follow and defects induced by compounds or mutations easy to observe (Kimmel et al., 1995). Finally, large numbers of eggs can be obtained, owing to their high fecundity, making zebrafish the perfect model for genetic studies and high-throughput compound screens (den Hertog, 2005; Wiley et al., 2017).

Zebrafish are frequently and intensively being used to investigate signaling in development and disease. For instance, bone morphogenetic protein receptor (BMPR) signaling is widely studied in zebrafish. Bone morphogenetic proteins (BMPs) are highly conserved secreted cytokines with key roles in organ formation and tissue homeostasis (Katagiri and Watabe, 2016). Depending on dose, BMPs induce different cell fates and control patterning within multicellular organisms during embryogenesis (Bier and De Robertis, 2015). A relatively easy to follow process in zebrafish involving BMP signaling is dorsoventral patterning. Knockout mutations introduced in distinct genes of the signaling cascade, including ligands, e.g. *bmp2* (also known as *bmp2a*) (Kishimoto et al., 1997), receptors, e.g. *activin receptor-like kinase* 2 (*alk2*) (Bauer et al., 2001) [in zebrafish also known as *alk8*, *lost-a-fin* or *acvrl1* (Mintzer et al., 2001)] or intracellular messengers, e.g. *smad5* (Kramer et al., 2002), all result in a dorsalization phenotype, including promotion of dorsal ectodermal cell fates at the expense of ventral tissues. Conversely, excess signaling caused by overexpression of Bmps, such as Bmp2 or Bmp7 (also known as Bmp7a) (Schmid et al., 2000), or loss of secreted Bmp antagonists, such as Chordin, Follistatin and Noggin (Dal-Pra et al., 2006), causes ventralization, the expansion of ventral tissue at the expense of dorsal structures. Overexpression of constitutively active Alk2 causes severe ventralization, whereas *alk2* null mutants show the opposite phenotype of dorsalization and loss of the ventral fin (Shen et al., 2009).

Overactive BMP signaling has been implicated in a plethora of human diseases. The most prominent example is the rare genetic disorder fibrodysplasia ossificans progressiva (FOP), in which fibrous tissue, such as muscles and ligaments, are progressively replaced by bone tissue (Pignolo and Kaplan, 2018). FOP patients carry a missense mutation in the gene encoding the BMPR activin A receptor type 1 (ALK2; also known as ACVR1) (Kaplan et al., 2009). The mutation in *ALK2* results in gain of function of ALK2. Despite great effort in recent years, there is currently no approved treatment for FOP (Pignolo and Kaplan, 2018). Moreover, *ALK2* is also found mutated in ∼25% of patients with the rare childhood brainstem tumor diffuse intrinsic pontine glioma (DIPG) (Taylor et al., 2014). In addition, more common diseases such as myeloid leukemia (Lefort and Maguer-Satta, 2020), chronic kidney disease (Kajimoto et al., 2015), vascular calcification (Derwall et al., 2012) and atherosclerosis (Saeed et al., 2012) are also linked to overactive BMP signaling. Zebrafish have been used to study the causal involvement of BMP signaling in a variety of skeletal and ocular diseases (Ye et al., 2009), including congenital FOP (LaBonty and Yelick, 2018; Mucha et al., 2018), radioulnar synostosis (Suzuki et al., 2020) and superior coloboma (Hocking et al., 2018). Targeting overactive BMP signaling for therapeutic gain has promise, but will require selective intervention.

BMPs exert their multifunctional effects on cells by interacting with selective cell surface type I and type II BMPRs, which are endowed with intracellular serine/threonine kinase domains. The type I receptors are also termed activin receptor-like kinases (ALKs). Upon BMP-induced type I/type II heteromeric complex formation, the constitutively active type II kinase phosphorylates the type I receptors on particular serine and threonine residues (Gomez-Puerto et al., 2019). Activated type I receptors promote phosphorylation of receptor-regulated SMAD1, SMAD5 and SMAD8 proteins, which act as transcription factor complexes by partnering with SMAD4. These heteromeric SMAD complexes translocate into the nucleus, where they interact in a DNA sequence-dependent manner with enhancers/promoters of target genes and regulate their expression (Hill, 2016). A typical target gene is *ID1*, and multimerizing the SMAD1/5 response elements in front of a minimal promoter generates a highly selective reporter system to interrogate BMP/SMAD signaling (Korchynskyi and ten Dijke, 2002). All four type I BMPRs [ALK1 (also known as ACVRL1), ALK2, ALK3 (also known as BMPR1A) and ALK6 (also known as BMPR1B)] activate the SMAD1/5 pathway. Ectopic expression of mutant, constitutively active type I BMPRs mimic the BMP signaling response. Type I receptors determine the signaling specificity in BMP-induced heteromeric complexes (Gomez-Puerto et al., 2019).

BMP inhibitors have been identified by small-molecule compound screens using zebrafish embryos. The first inhibitor to be identified was dorsomorphin, which induces developmental defects that phenocopy BMP mutants (Yu et al., 2008). Dorsomorphin targets type I BMPRs and, moreover, rescues the phenotype caused by overexpression of constitutively active Alk2 in zebrafish (Shen et al., 2009; Yu et al., 2008). Unfortunately, dorsomorphin also harbors off-target effects, such as inhibition of vascular endothelial growth factors (Vegfs) and AMP-activated protein kinase (Ampk) (Cannon et al., 2010; Hao et al., 2010; Zhou et al., 2001). However, related BMP inhibitors such as DMH-1 and LDN-193189, sharing the same pyrazolo[1,5-a]pyrimidine core as dorsomorphin, have fewer off-target effects and are potently targeting type I BMPR kinases, predominately Alk2 (Hao et al., 2010). Finally, more recent phenotype-based zebrafish embryo screens led to the discovery of other classes of small-molecule inhibitors acting in the BMP pathway (Cheng et al., 2019; Dasgupta et al., 2017; Gebruers et al., 2013; Sanvitale et al., 2013).

Previously, we performed a large-scale screen of over 10,000 fungal filtrates on developing zebrafish embryos (Hoeksma et al., 2019). Embryos treated with fungal filtrate were compared to an untreated control and filtrates were scored as positive if any developmental defects were observed at 48 h post-fertilization (hpf). Here, we describe the identification of cercosporamide from one of these fungi, which induced developmental defects reminiscent of BMP inhibition. We demonstrate that cercosporamide inhibited BMP/SMAD signaling in mammalian cells and zebrafish embryos. Moreover, using kinase assays with purified kinases, cercosporamide was found to be a direct inhibitor of BMPR type I kinase activity. Our results indicate that cercosporamide is a bona fide BMPR type I inhibitor in zebrafish and mammalian cultured cells.

## RESULTS

### Purification and identification of cercosporamide

**Fig. 1.**
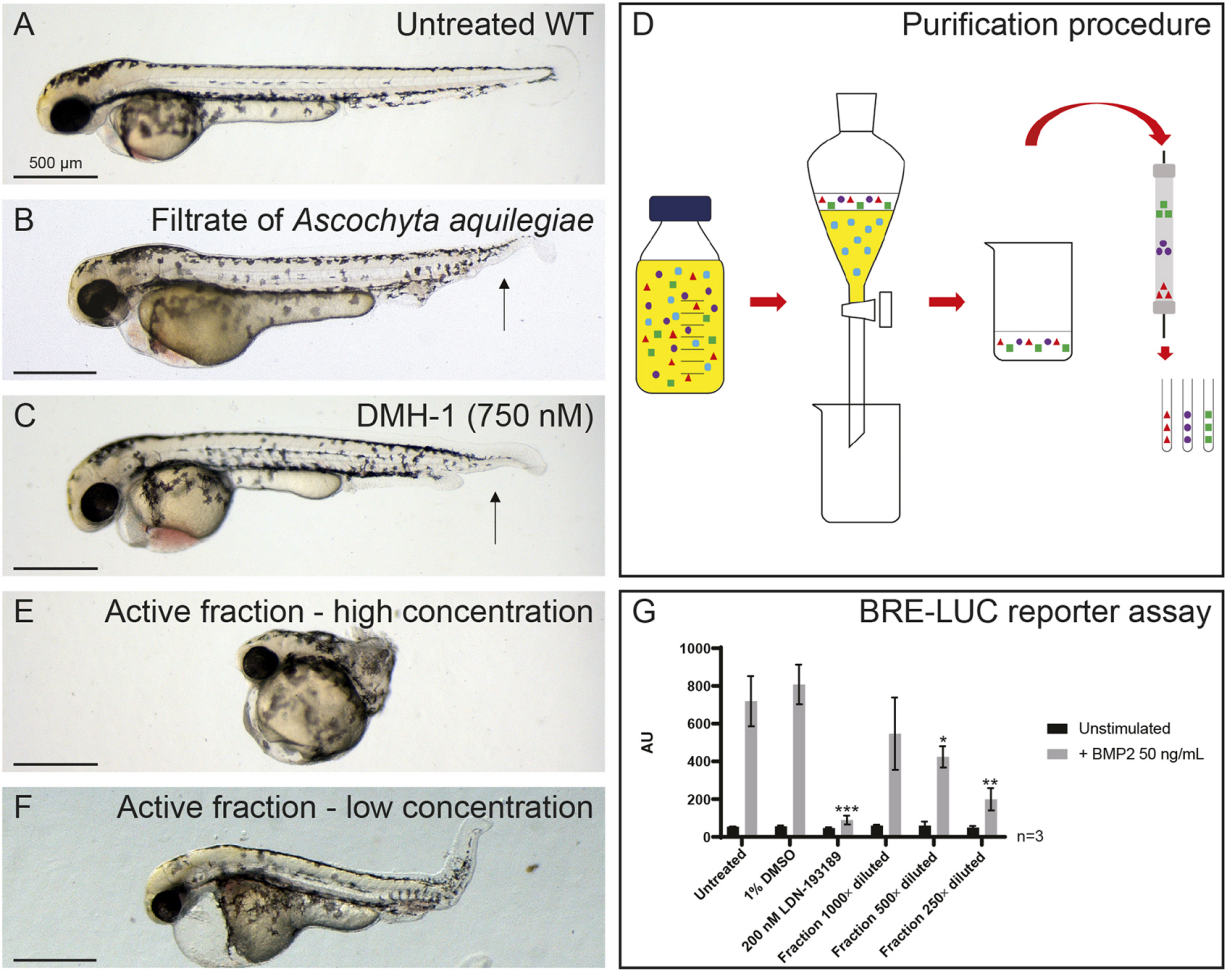
**Identification of a fungal filtrate with activity consistent** **with inhibition of BMP signaling.** (A) Untreated control, not treated with fungal filtrate. WT, wild type. (B) An example of a phenotype induced by the filtrate of *A. aquilegiae* mixed in 1:1 ratio with E3 medium, with treatment at 6-48 hpf. (C) Phenotype induced by 750 nM DMH-1. The arrows in B and C indicate loss of the ventral fin. (D) Schematic overview of purification of active component. The filtrate is extracted with 3×1/3 volume ethyl acetate. The ethyl acetate fractions are than combined and dried. The residue is dissolved in DMSO and subsequently loaded onto a preparative HPLC column. Fractions are collected every 63 s. (E,F) Phenotypes induced by purified fraction, at high (E) and low (F) concentrations. In A-C,E,F, ten embryos were incubated per condition; representative pictures are shown. (G) Dose-dependent inhibitory effects of purified fraction of fungal filtrate on BMP2-induced Smad1/5-dependent BRE-luc transcriptional reporter activity. Lack of significant effect of vehicle control DMSO and potent antagonizing effect of BMPR type I kinase inhibitor LDN-193189 are shown. Results are expressed as mean±s.d., *P<0.05, **P<0.01, ***P<0.001 (Student’s *t*-test, two-tailed, unpaired).

In a screen of over 10,000 fungal filtrates on developing zebrafish embryos, we found that the filtrate of *Ascochyta aquilegiae* (CBS 168.70) induced characteristic defects, including lack of the ventral fin at the posterior part of the tail at 48 hpf. In addition, several embryos displayed the formation of secondary tissue, which was not observed in the non-treated control (Fig. 1A,B). This phenotype is strikingly similar to the phenotype induced by known BMPR type I kinase inhibitors such as dorsomorphin, LDN-193189 and DMH-1 (Fig. 1C), and to BMP mutants previously reported in multiple studies (Gebruers et al., 2013; Yang and Thorpe, 2011; Yu et al., 2008).

To identify the active compound in the fungal filtrate, we generated 5 l of filtrate and performed activity guided purification (Fig. 1D). First, we performed liquid-liquid extraction and tested the resulting products. We established successful extraction of the active components as the extract induced a similar phenotype as the filtrate. Next, we fractionated the extract using preparative high-performance liquid chromatography (HPLC) and tested the consequent fractions in the zebrafish phenotypic assay. One fraction induced a severely truncated phenotype in zebrafish embryos, which upon dilution turned out to induce a similar phenotype as the extract (Fig. 1E,F) and hence contained the active compound(s).

Next, we tested the active fraction for effect on a BMP2-induced SMAD1/5-dependent transcriptional reporter (BRE-luc) assay in HepG2 hepatocellular carcinoma cells. We found that the compound dose dependently inhibited BMP2-induced BRE-luc activity, like the BMPR kinase inhibitor LDN-193189 (Fig. 1G). Taken together, these results strongly suggest that the active fungal preparation inhibited BMP signaling in zebrafish embryos and human cells.

**Fig. 2.**
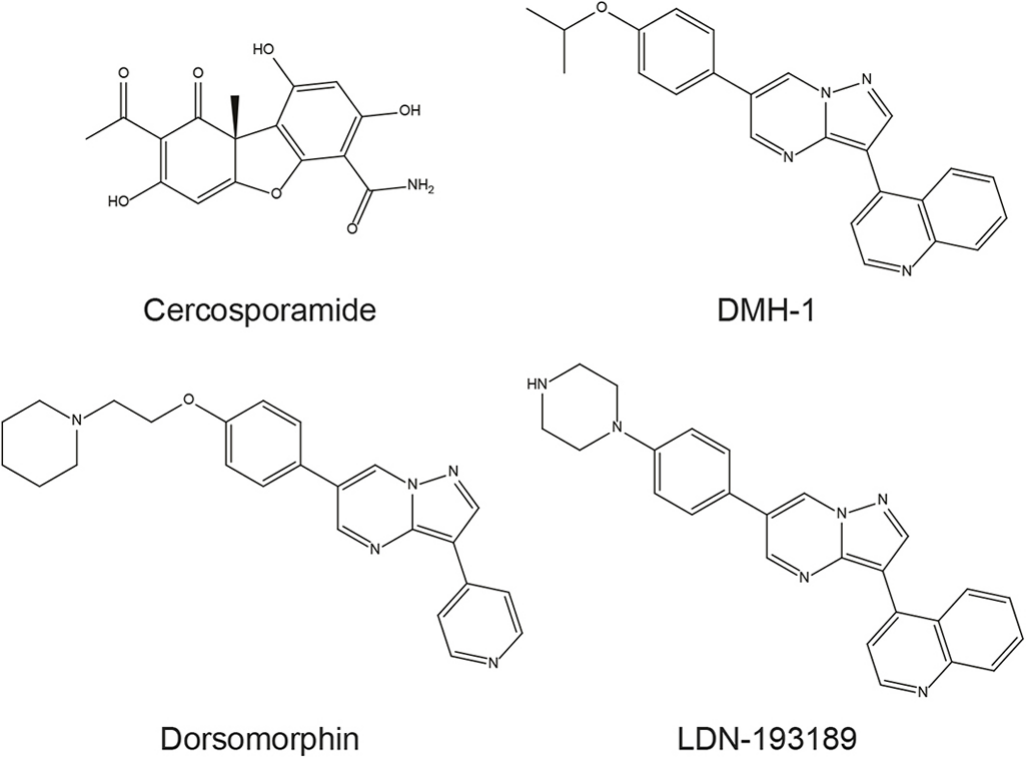
**Molecular structures of cercosporamide and established BMP inhibitors dorsomorphin, DMH-1 and LDN-193189.**

**Fig. 3.**
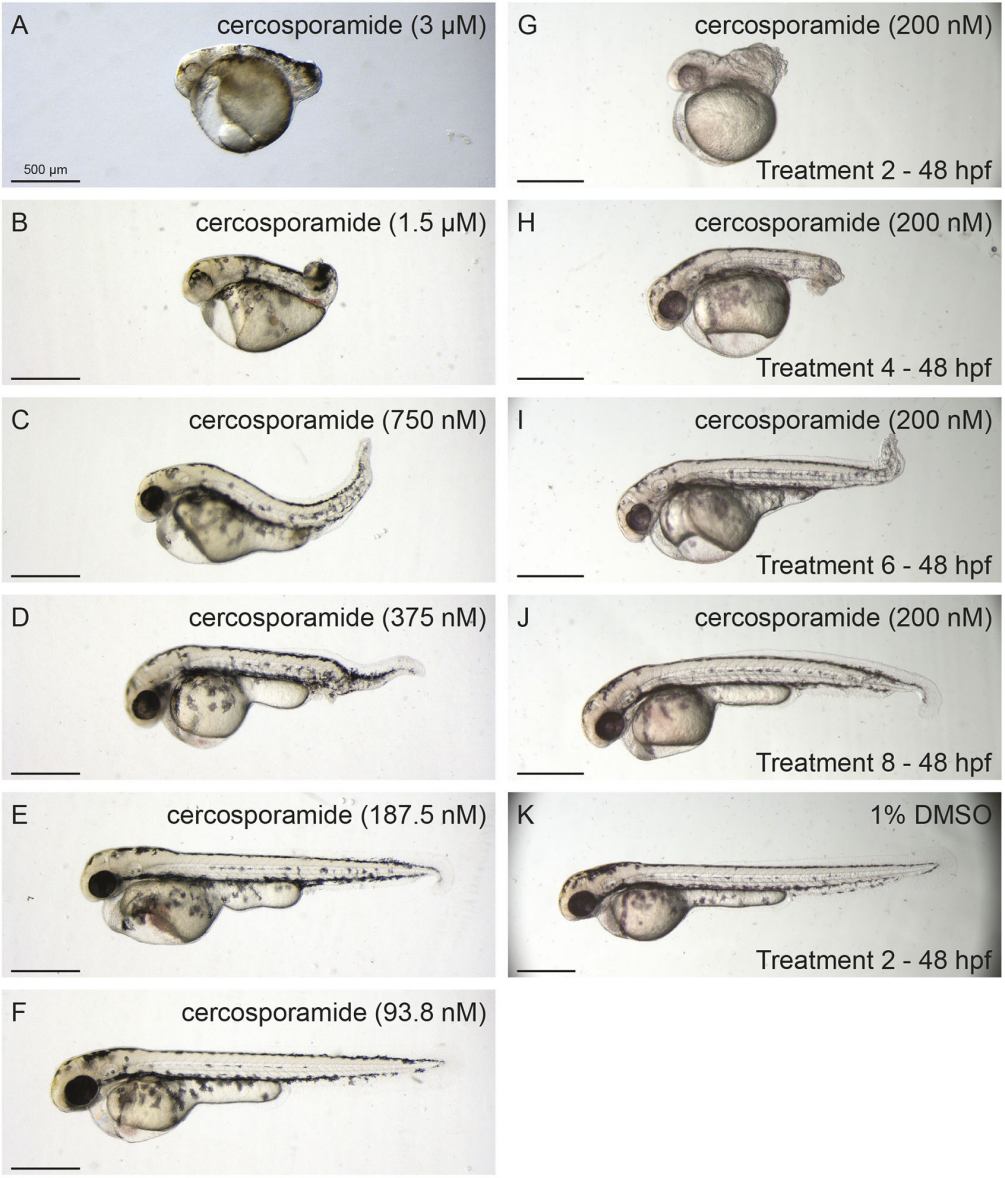
**Dose- and time-dependent developmental defects of cercosporamide in zebrafish embryos.** (A-F) Examples of phenotypes caused by a cercosporamide dilution range of 3 µM to 93 nM. (G-J) Examples of phenotypes caused by 200 nM cercosporamide with different treatment start times as indicated in hpf. (K) Control, 1% DMSO-treated embryo. Ten embryos were incubated per condition; representative pictures are shown.

Subsequently, the active fraction was tested via analytical HPLC for purity, and the diode array detection allowed us to obtain an ultraviolet (UV)-visible (Vis) spectrum with maximum absorbance (λ_max_) at 223 nm and 257 nm and a shoulder peak (sh) at 310 nm (Fig. S1). High-resolution mass spectrometry (HRMS) of the active compound revealed a mass of 332.0765, which suggested several options for a molecular formula. Finally, the remainder of the fraction was dried and used for nuclear magnetic resonance (NMR) spectroscopy. The resulting spectrum (Fig. S2) very closely matched data on cercosporamide (Fig. 2) reported by Sussman et al. (2004), which were also consistent with the accurate mass measurement of 332.0765. To confirm definitively that the compound we found to induce the BMP inhibitor phenotype was cercosporamide, we obtained commercially available cercosporamide and verified its activity in zebrafish embryos (Fig. 3). In addition, we established that commercially available cercosporamide eluted from the analytical HPLC column at a similar retention time as the purified compound. In all further experiments, commercially available cercosporamide was used to characterize its activity. Cercosporamide: C_16_H_13_NO_7_. HRMS: found 332.0765 (M+H), calculated 332.0770 for C_16_H_14_NO_7_. NMR (400 MHz, d_6_-DMSO): 13.55 (s, 1H); 10.56 (s, 1H); 8.25 (s, 1H); 7.54 (s, 1H); 6.22 (s, 1H); 6.14 (s, 1H); 2.57 (s, 3H); 1.73 (s, 3H) (Fig. S1). UV-Vis λ_max_: 223 nm, 257 nm, 310 nm (sh).

### Biological activity of cercosporamide in zebrafish assays

The biological activity of cercosporamide was tested using a dilution range in our zebrafish assay from 30.2 µM (10 µg/ml) downwards. Treatment started at 7 hpf and was continuous until effects were observed at 48 hpf. Cercosporamide treatment was lethal above 3 µM. At 3 µM and 1.5 µM, cercosporamide induced a severely truncated phenotype (Fig. 3A,B). Upon dilution of commercially available cercosporamide, the loss-of-ventral-fin phenotype became evident (Fig. 3C-E), similar to the phenotype induced by the purified fraction from *A. aquilegiae* (Fig. 1F). Further dilution abolished the effect of cercosporamide at 100 nM (Fig. 3F). Similarly, dose-dependent effects were observed with DMH-1, although it appeared that the treatment window for DMH-1 was narrower than that for cercosporamide (Fig. S3). Furthermore, we tested the effect of starting treatment at different time points using 200 nM cercosporamide and 2-h intervals. Starting treatment at 2 hpf induced a severely truncated phenotype, comparable to treatment with 1.5 µM from 7 hpf onwards (Fig. 3G). The effect of 200 nM cercosporamide decreased dramatically when treatment was started at later time points, until only a mild phenotype was induced when treatment started at 8 hpf (Fig. 3H-K). Treatment of zebrafish embryos with DMH-1 at 8 hpf also only induced mild defects (Fig. S3), indicating that, for the characteristic ventral fin defects to occur at 48 hpf, treatment of the embryos with BMP inhibitors had to start before 8 hpf.

**Fig. 4.**
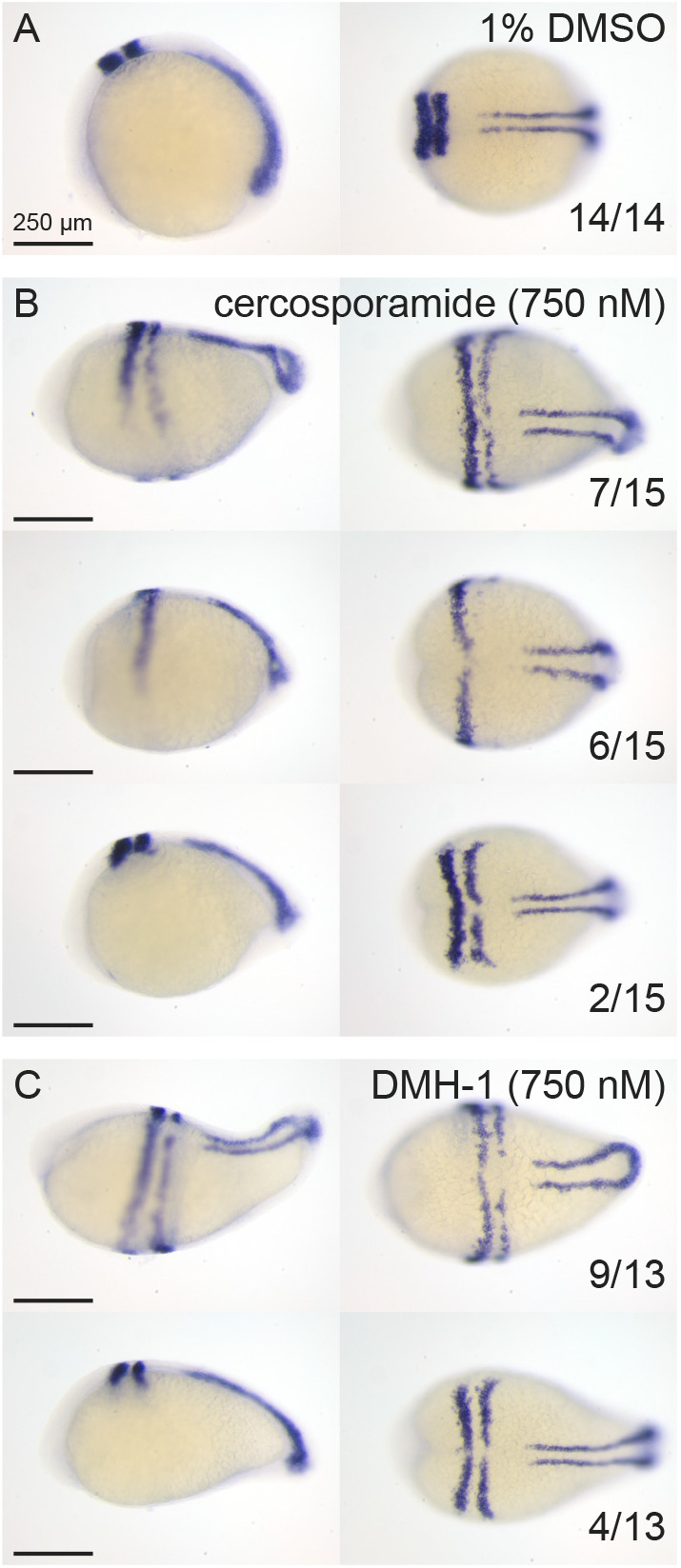
***In situ* hybridization using *krox20/myod*-specific probes confirms that cercosporamide and the well-known BMP inhibitor DMH-1 induced similar defects in zebrafish development.** (A-C) Embryos were treated with 1% DMSO (control) (A), 750 nM cercosporamide (B) or 750 nM DMH-1 (C) from 6 hpf onwards and fixed at 12 hpf. *In situ* hybridization was performed using *krox20/myod*-specific probes. Representative examples of resulting embryos are shown with lateral view on the left and dorsal view on the right. In the bottom-right corner, the fraction of embryos showing the pattern is depicted.

The phenotypes induced by cercosporamide in zebrafish embryos were remarkably similar to known BMPR type I kinase inhibitors, although cercosporamide is structurally distinct and does not contain the same pyrazolo[1,5-a]pyrimidine core (Fig. 2). To compare the activity of cercosporamide to known BMPR type I kinase inhibitors, we performed additional experiments. First, similar to Yu et al. (2008), we fixed embryos treated with cercosporamide or DMH-1 at 12 hpf and performed *in situ* hybridization using *krox20* (also known as *egr2a*)*-* and *myod* (also known as *myod1*)-specific probes, which stain rhombomeres 3 and 5 and the presomitic mesoderm, respectively (Oxtoby and Jowett, 1993; Weinberg et al., 1996). Together, these are well-established markers for convergence and extension cell movements in the developing zebrafish embryo. Both cercosporamide and DMH-1 induced lateral expression of *krox20* and a more oval shape of the embryo compared to the DMSO treated control (Fig. 4). *myod* expression was not affected at these early stages. These results are consistent with the effects of dorsomorphin on early-stage zebrafish embryos (Yu et al., 2008) and with developmental defects observed in BMP pathway mutants (Little and Mullins, 2004).

**Fig. 5.**
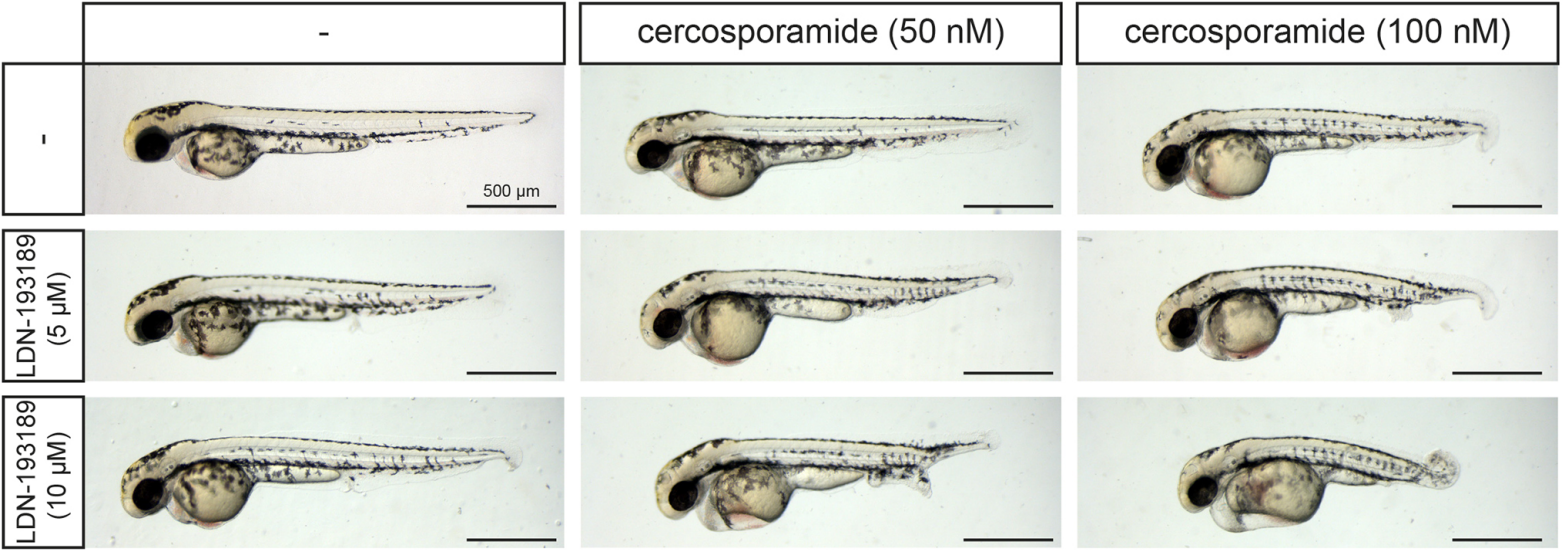
**Cercosporamide and known BMP inhibitors cooperate.** Combination treatments of zebrafish embryos suggest that cercosporamide acts on the BMP signaling pathway. Embryos were treated with cercosporamide (50 nM or 100 nM), LDN-193189 (5 µM or 10 µM) or combinations from 7 hpf onwards as indicated. Ten embryos were incubated per condition; representative pictures of treated embryos are shown.

To assess whether cercosporamide and known BMP inhibitors exert their effects by inhibition of the same signaling pathway, we investigated whether cercosporamide and LDN-193189 cooperate by treatment of zebrafish embryos with combinations of low concentrations of cercosporamide and LDN-193189. When tested separately, 50 nM cercosporamide and 5 µM LDN-193189 did not induce detectable developmental defects. However, when tested in combination, these low concentrations induced a partial loss of the ventral fin (Fig. 5), suggesting that cercosporamide and LDN-193189 act on the same pathway. Combination treatments using different concentrations of cercosporamide and LDN-193189 always induced a more severe phenotype than did the single treatments (Fig. 5). Similar results were obtained when combining cercosporamide with either dorsomorphin or DMH-1 (Fig. S4). Based on these results, we conclude that cercosporamide can exert inhibitory effects on the BMP pathway.

### Cercosporamide inhibits BMP-induced responses in mammalian cells

**Fig. 6.**
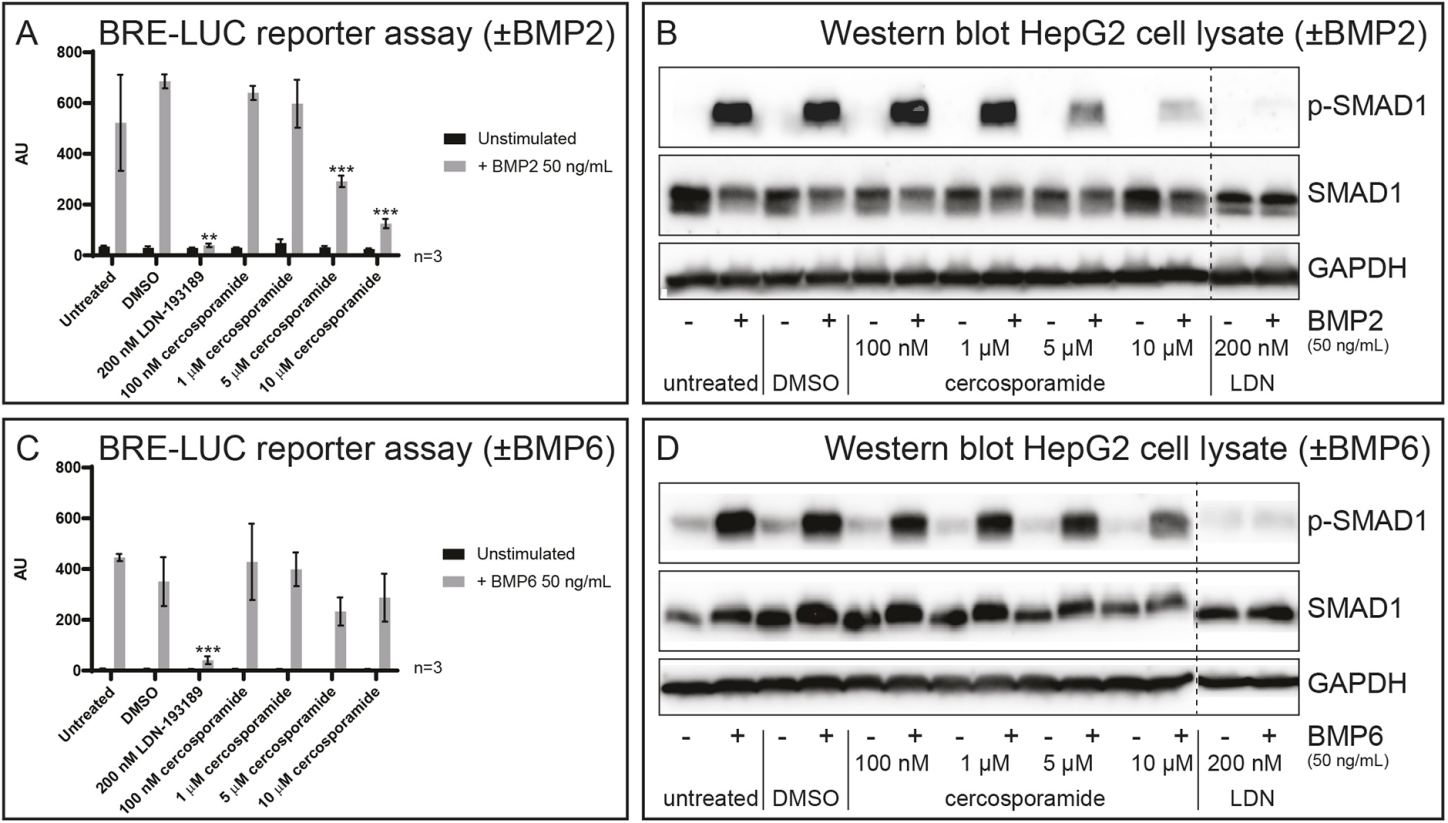
**Cercosporamide inhibits BMP/SMAD signaling in mammalian cells.** (A) HepG2 cells with BRE-luc reporter were treated with BMP2 (50 ng/ml) or not treated (−). Control (1% DMSO), LDN-193189 (200 nM) or a range of concentrations of cercosporamide (100 nM to 10 µM) were added and luciferase activity was determined. Averages of triplicate measurements are depicted as arbitrary units. (B) HepG2 cells were treated with BMP2 (50 ng/ml) or not (−), and with 1% DMSO (control), a range of concentrations of cercosporamide (100 nM to 10 µM) or LDN-193189 (200 nM). Cells were lysed and the lysates run on SDS-PAGE gels. The material on the gel was transferred to blots and parallel blots were probed using antibodies, specific for phosphoSMAD1/5/8 (p-SMAD1) and SMAD1 or GAPDH (loading control). Detection was performed by enhanced chemiluminescence (ECL). Representative blots are shown. (C) As in A, except BMP6 (50 ng/ml) was used instead of BMP2. (D) As in B, except BMP6 (50 ng/ml) was used instead of BMP2. **P<0.01 and ***P<0.001 (Student’s *t*-test, two-tailed, unpaired). All samples in B and D were run on the same gel; the dashed lines indicate where the blots were cut.

Next, we examined the effects of cercosporamide on BMP signaling in mammalian cells. We specifically examined the inhibition of signaling through BMP2, ligand of type I receptors ALK3 and ALK6, and BMP6, ligand of ALK2. First, we performed a luciferase assay measuring BMP2-induced LUC expression in transfected HepG2 cells after treatment with cercosporamide (Fig. 6A). We included LDN-193189 as a positive control and dimethyl sulfoxide (DMSO) as a vehicle control. Cercosporamide inhibited the BMP2-induced response in a dose-dependent manner, although not as potently as LDN-193189. Subsequently, we investigated the effect of cercosporamide on BMP2-induced SMAD1/5 phosphorylation in HepG2 cells. Comparable to the luciferase assay, high concentrations of cercosporamide are capable of blocking SMAD1/5 phosphorylation, although not to same extent as 200 nM LDN-193189 (Fig. 6B). Finally, we also assessed the effect of cercosporamide on BMP6-induced BRE-reporter activity and SMAD1/5 phosphorylation. Surprisingly, we observed only minor inhibition of SMAD1/5 phosphorylation at the higher cercosporamide concentrations (Fig. 6C,D). These results demonstrate that cercosporamide inhibited BMPR-induced signaling in mammalian cells.

### Cercosporamide is a direct BMPR type I kinase inhibitor

**Fig. 7.**
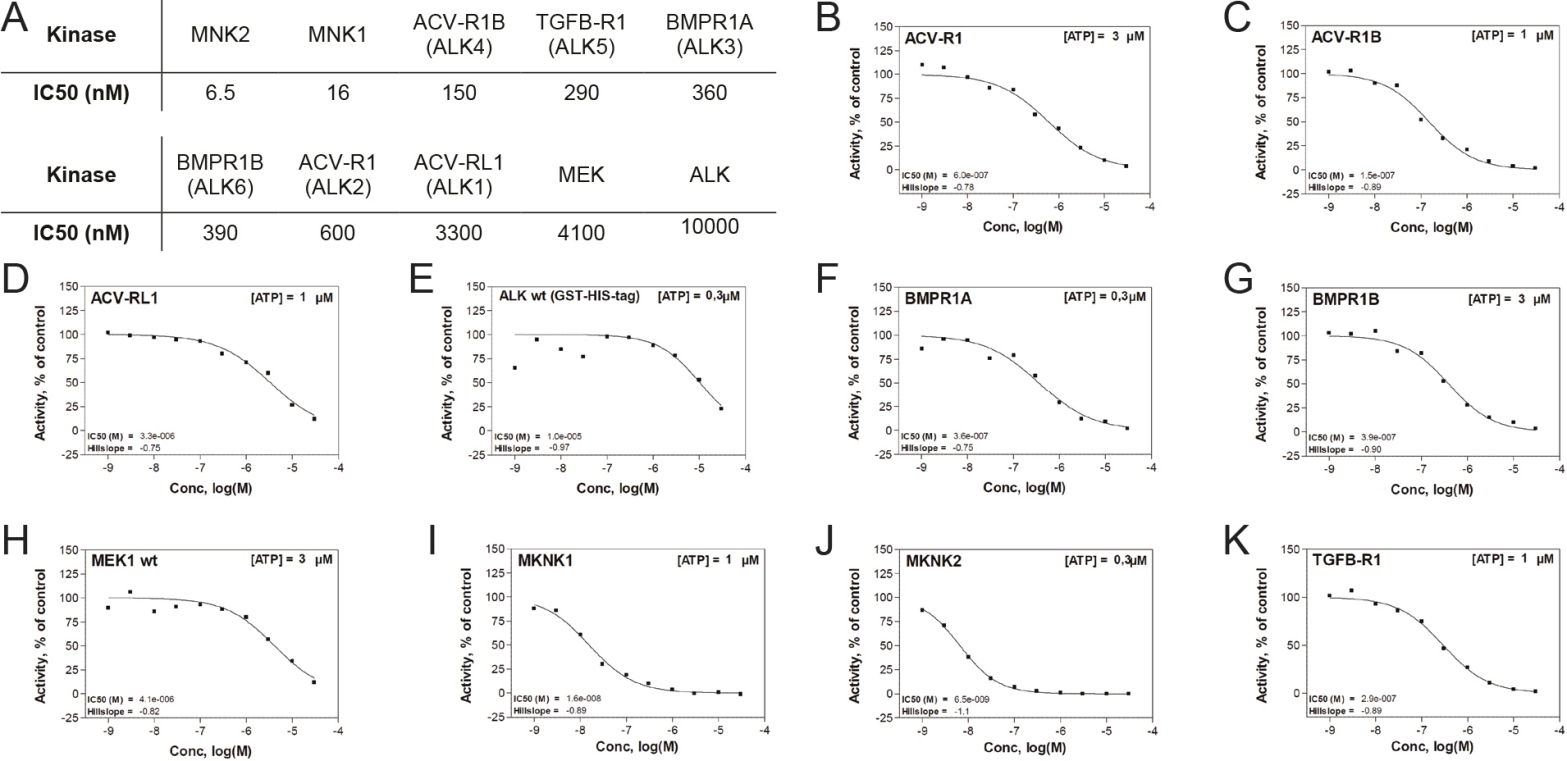
**Cercosporamide inhibits kinase activity of purified ALK receptors *in vitro*.** (A) IC50 values of cercosporamide-mediated inhibition of a panel of ten kinases. (B-K) Activity graphs from which the IC50 values in A were derived. The kinases include six type I ALK BMPRs, mitogen-activated protein kinase (MAPK)-interacting protein kinases 1 and 2 (MNK1, MNK2), mitogen-activated protein kinase kinase (MEK) and the tyrosine kinase, anaplastic lymphoma kinase (ALK). wt, wild type.

Cercosporamide may inhibit BMP signaling by inhibition of ligand-receptor interaction, by inhibiting receptor activity or by inhibiting downstream SMAD phosphorylation. Cercosporamide is reported to have serine/threonine kinase inhibitor activity with strong inhibitor activity on the cytoplasmically localized kinases, MNK1 and MNK2 (also known as MKNK1 and MKNK2) (Altman et al., 2018; Konicek et al., 2011; Sussman et al., 2004). To assess whether type I BMPRs were inhibited by cercosporamide, a radiometric protein kinase activity assay was performed using ALK1-ALK6. All ALKs, except for ALK1, showed a half-maximal inhibitory concentration (IC50) in the nanomolar range, indicating that cercosporamide might indeed act through direct inhibition of ALKs. As a control, inhibition of MNK1 and MNK2 was assessed. Indeed, MNK1 and MNK2 were strongly inhibited by cercosporamide, with IC50 values of 16 nM and 6.5 nM, respectively (Fig. 7). As a negative control, we included BMP signaling-unrelated kinases MEK and a tyrosine kinase, anaplastic lymphoma kinase (ALK), which were indeed 7- to 67-fold less sensitive to cercosporamide than the ALK serine/threonine kinases (Fig. 7).

Given the strong inhibitory activity of cercosporamide on MNK1 and MNK2, we wondered whether the effects of cercosporamide might be due to inhibition of MNK1 and/or MNK2. To test this, we investigated the effect of another MNK inhibitor, eFT508, on zebrafish and cultured HepG2 cells. At the maximum tested concentration of 20 µM, no effect was observed on zebrafish embryo development or on SMAD1/5 phosphorylation in HepG2 cells (Fig. S5), suggesting that inhibition of MNK1 and/or MNK2 was not involved in the observed effects of cercosporamide. Together, our data are consistent with cercosporamide affecting zebrafish embryo development and mammalian cultured cells by direct inhibition of BMPR type I kinase activity.

### Cercosporamide inhibits caALK signaling in mammalian cells and rescues caAlk2-induced developmental defects in zebrafish embryos

**Fig. 8.**
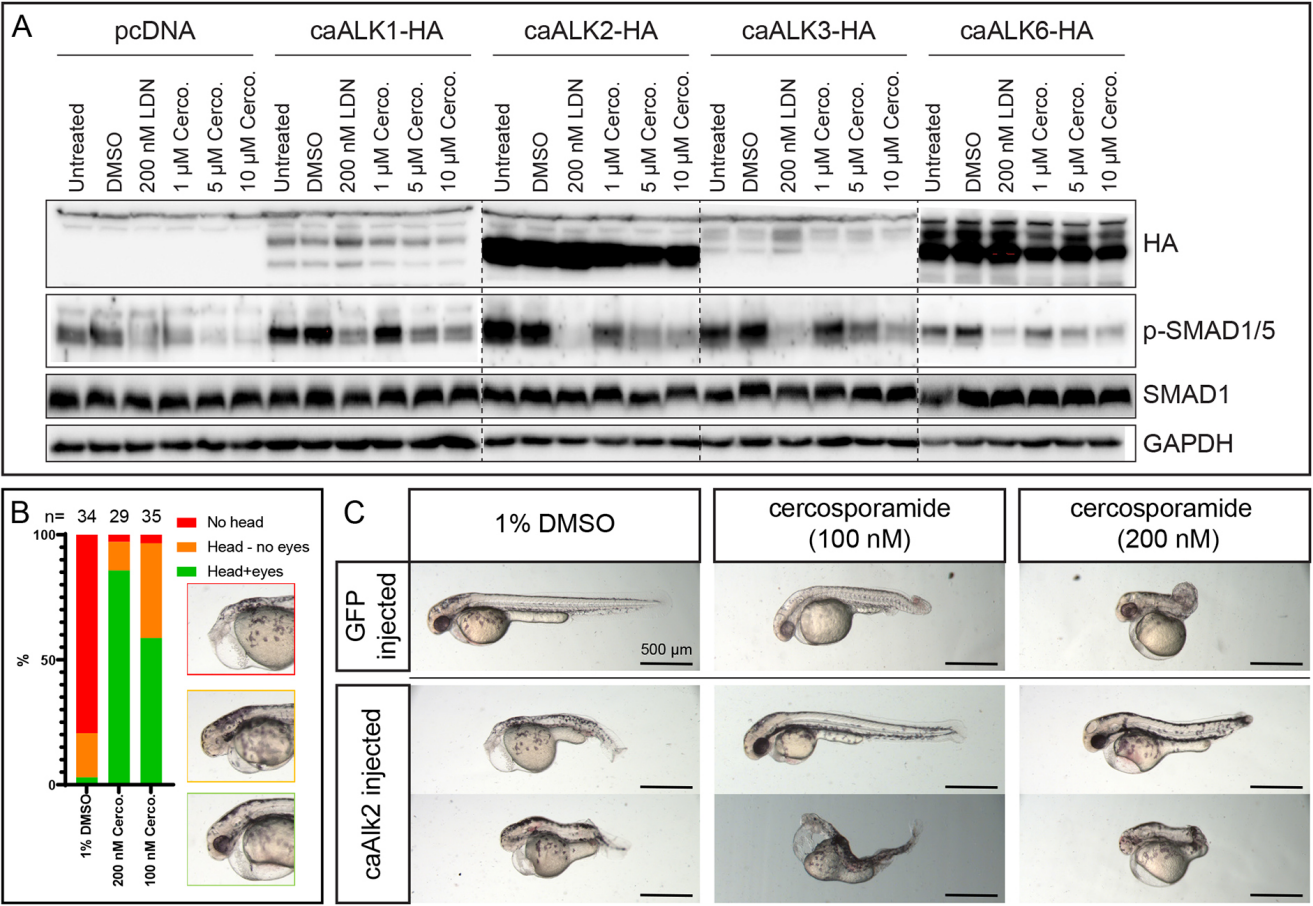
**Cercosporamide selectively inhibits caBMPR type I in cultured mammalian cells and zebrafish embryos.** (A) HEK 293T cells were transfected with empty vector (pcDNA) or expression vectors for caALK1, caALK2, caALK3 or caALK6 each with a haemagglutinin (HA) epitope tag at the carboxy terminus. The cells were treated with vehicle (1% DMSO), LDN193189 (200 nM) or a range of cercosporamide concentrations (1-10 µM as indicated). Subsequently, the cells were lysed and the lysates run on SDS-PAGE gels. The material on the gel was transferred to blots and parallel blots were probed with antibodies specific for HA (epitope tag on receptors), phosphoSMAD1/5/8 (p-SMAD1), SMAD1 or GAPDH (loading control). Dashed lines indicate the borders of different gels. (B) Cercosporamide partially rescued caAlk2-induced developmental defects in zebrafish embryos *in vivo*. Bar chart shows the phenotype distribution of embryos injected with caAlk2 mRNA and subsequently treated with 1% DMSO, 100 nM or 200 nM cercosporamide from 2 hpf onwards. The severity of the phenotype (examples depicted in the insets) is plotted as red (severe; no head), orange (intermediate; head structures present, no eyes) and green (mild; head structure with eyes detectable). The total number of embryos (*n*) is indicated. (C) The phenotypes of the rescued embryos are highly variable; therefore, we depicted two representative individuals of ten embryos that were treated for each condition.

Constitutively active (ca) ALKs have been generated that activate downstream signaling in a ligand-independent manner. To investigate the effect of cercosporamide on different BMPRs type I in living cells, we ectopically expressed caALK1, caALK2, caALK3 or caALK6 in human embryonic kidney (HEK) 293T cells. Despite varying expression levels, all type I caBMPRs induced SMAD1/5 phosphorylation, albeit to different extents, which was strongly reduced by treatment with the BMP inhibitor LDN-193189 (Fig. 8A). Cercosporamide inhibited SMAD1/5 phosphorylation in response to each of these caALKs in a dose-dependent manner (Fig. 8A). We also investigated the effect of cercosporamide on caALK5, constitutively active transforming growth factor-β (TGF-β) type I receptor (TGFBR1). Surprisingly, caALK5-induced SMAD1/5 phosphorylation was only weakly inhibited by cercosporamide (Fig. S6), even though ALK5 kinase activity was potently inhibited by cercosporamide *in vitro* (Fig. 7). To further investigate the role of ALK5 in the effects of cercosporamide, we tested the effects of two selective ALK5 kinase inhibitors, i.e. ALK5 kinase inhibitor II and A83-01, on zebrafish embryos. Surprisingly, both these compounds induced a severely curved tail and fused eyes in a broad concentration range (Fig. S6). These developmental defects are distinct from the phenotype induced by cercosporamide, suggesting that cercosporamide does not act *in vivo* through inhibition of Alk5. Thus, cercosporamide selectively inhibits type I caBMPRs, but not caTGF-β-like receptors, in cultured cells and zebrafish embryos.

Next, we examined the ability of cercosporamide to inhibit Alk2 *in vivo* in zebrafish embryos expressing caAlk2. mRNA encoding caAlk2, combined with GFP mRNA was injected at the one-cell stage. The embryos were incubated with either 100 nM or 200 nM cercosporamide or 1% DMSO (vehicle control) from 2 hpf onwards. As a control, we injected GFP mRNA only. We observed a variety of phenotypes in caAlk2-injected embryos treated with DMSO, which we categorized in three groups (Fig. 8B). We observed an absence of a head in 79% of the cases when treated with DMSO (Fig. 8B,C). Furthermore, in this group, 18% of the embryos developed a head, but no eyes were developed. Only 3% of the embryos developed a seemingly normal head. Treatment with cercosporamide largely rescued the head phenotype in a dose-dependent manner, in that 86% and 58% developed a head with eyes, following treatment with 200 nM and 100 nM, respectively (Fig. 8B). Conversely, the cercosporamide-induced developmental defects in the posterior region of the control GFP-injected embryos were not completely rescued by caAlk2 injection, although there appeared to be some improvement. Overall, cercosporamide partially rescued the effects of caAlk2 injection and caAlk2 injection partially rescued the posterior defects induced by cercosporamide, indicating that cercosporamide is an inhibitor of Alk2 *in vivo*.

## DISCUSSION

Here, we describe the purification and identification of cercosporamide from *A.* *aquilegiae*. The biological activity in developing zebrafish embryos suggested that cercosporamide is an inhibitor of Bmp signaling. Subsequent analyses indicated that cercosporamide inhibited the kinase activity of type I BMPRs *in vitro*, inhibited BMPR type I signaling in mammalian cells and rescued the caALK2-induced developmental defects in zebrafish embryos *in vivo*.

Cercosporamide is a known secondary metabolite of fungi. Previously, it was purified and identified from a strain of the fungus *Cercosporidium henningsii* (Sugawara et al., 1991), and has further been found to be produced by fungal strains of the *Lachnum* and *Pseudaegerita* genus (Hosoya et al., 2011). This is the first time that cercosporamide is described as a metabolite from *A. aquilegiae*.

Initially, cercosporamide was considered to be a potent antifungal agent and phytotoxin (Sugawara et al., 1991). Later, it was shown that cercosporamide inhibits PKC1 in yeast (Sussman et al., 2004). Furthermore, cercosporamide has been tested on a panel of kinases and was identified as a potent MNK1 and MNK2 inhibitor. Inhibition of MNK1 and MNK2 kinases could be the underlying mechanism for cercosporamide-mediated suppression of growth of human hepatocellular carcinoma and acute myeloid leukemia precursors (Altman et al., 2018; Konicek et al., 2011; Liu et al., 2016). It is noteworthy that ALK4 (also known as ACVR1B) was also included in the panel of kinases and was found to be inhibited by cercosporamide (Konicek et al., 2011), consistent with our results (Fig. 7). However, this observation has not been pursued any further and, at the time, no functional assays were performed to test the hypothesis that cercosporamide exerts its function through inhibition of TGF-β family type I receptor kinases.

Using the zebrafish embryo model, we identified cercosporamide as a potent BMP inhibitor. Cercosporamide mimics the phenotype induced by established BMP inhibitors and the phenotype observed in zebrafish mutants with loss-of-function mutations in factors of the BMP signaling pathway. Moreover, we showed that cercosporamide rescued developmental defects induced by caALK2 overexpression in a similar manner to DMH-1. Cercosporamide also inhibited BMP signaling in human cells. It is evident that there is a difference in the concentrations of cercosporamide and established small-molecule BMP inhibitors that are needed to induce developmental defects in zebrafish embryos and human cell lines. This difference might be caused by species differences (zebrafish versus human) or differences in the model used (embryo versus cell line). The mechanism of action of cercosporamide remains to be determined definitively. The molecular structure of cercosporamide is completely different from the structure of established BMP inhibitors, which mostly contain a pyrazolo[1,5-a]pyrimidine core. Hence, our results with cercosporamide could unlock an entirely different class of molecules that can be used as BMP inhibitors, potentially through a distinct working mechanism. Future follow-up analyses to elucidate the interaction of cercosporamide with its targets at the structural and molecular level will provide insight into the mode of action of cercosporamide. Our work clearly underlines the value of performing small-molecule compound screens on zebrafish embryos in order to uncover potential new drugs.

Our data are consistent with cercosporamide acting through inhibition of ALK2. However, ALK2 was not the most potently inhibited BMPR in the *in vitro* kinase assays (Fig. 6). ALK4 and ALK5 were inhibited more efficiently, consistent with published data (Konicek et al., 2011). However, cercosporamide induced different developmental defects than renowned Alk5 inhibitors in zebrafish (Fig. S7). In the reporter assays, ALK3 and ALK6 appeared to be more potently inhibited than ALK2. We cannot exclude the possibility that Alk3 and Alk6 are involved in the cercosporamide-induced developmental defects. However, the developmental defects induced by cercosporamide in zebrafish embryos have a striking similarity to zebrafish mutants that lack functional Alk2 (Bauer et al., 2001; Mintzer et al., 2001). Moreover, caAlk2-induced developmental defects were rescued by cercosporamide in zebrafish embryos *in vivo*. It appears that the *in vivo* effects of cercosporamide not only depend on the specificity for distinct ALKs, but also on other factors that determine the function of the different ALKs in development. *In vivo*, cercosporamide might exert its effects mainly through inhibition of Alk2.

Taken together, our results suggest that cercosporamide and possibly derivatives of cercosporamide have the potential to be used as BMP inhibitors, thus unlocking a new class of molecules that may be developed further for use in a clinical setting; for instance, to combat diseases with overactive BMPR signaling, including FOP and DIPG.

## MATERIALS AND METHODS

### Zebrafish embryo assay

Zebrafish eggs obtained from family crosses of Tuebingen long fin zebrafish lines were used to assess the biological activity of all samples. The eggs were washed with fresh E3 medium and subsequently divided over 24-well plates, with ten embryos per well in 1000 µl E3 medium. Samples were added to the wells at various times as mentioned in the figures and legends. At 48 hpf, the zebrafish embryos were inspected for morphological developmental defects. Embryos displaying morphological defects were imaged using a Leica MZFLIII microscope equipped with a Leica DFC320 camera or Leica M165 FC microscope equipped with a Leica DMC5400 camera.

All procedures involving experimental animals were approved by the local animal experiments committee (Koninklijke Nederlandse Akademie van Wetenschappen-Dierexperimentencommissie) and performed according to local guidelines and policies in compliance with national and European law. Adult zebrafish were maintained as previously described (Aleström et al., 2019).

### Culture and isolation of active compound

Initially, the fungus *A.* *aquilegiae* (CBS 168.70) was grown on a cornmeal agar plate for 7 days at 25°C. The plate with mycelium was then cut into cubes of approximately 5×5 mm. Subsequently, a bottle of 100 ml containing 50 ml Czapek Dox Broth+0.5% yeast extract was inoculated with two cubes and incubated at room temperature for 7 days. The medium was filter sterilized using a 0.45 µm Millipore filter and tested in serial dilution in the zebrafish embryo assay. In order to increase the yield of the active components, we optimized the growth conditions for this fungus before generating a large batch of filtrate. Ultimately, we inoculated 100 bottles containing 50 ml Czapek Dox Broth without the addition of yeast extract and incubated the medium at 15°C for 14 days. The medium was filtered as mentioned above in batches of 1 l each.

Subsequently, each liter was extracted with 3× ±300 ml ethyl acetate. The ethyl acetate was combined and evaporated using a rotation evaporator. The residue was dissolved in 2 ml DMSO, of which a small aliquot was used in the zebrafish embryo assay to verify the successful extraction of the active components. Successively, the extract was fractionated on a modular preparative HPLC system, consisting of a Shimadzu CBM-20A controller, a Shimadzu LC-20AP pump and a Shimadzu FRC-10A fraction collector using a C18 reversed-phase Reprosil column (10 µm, 120 Å, 250×22 mm) and a Shimadzu SPD-20A UV light detector set at 214 nm and 254 nm. The mobile phase was 0.1% trifluoroacetic acid in acetonitrile:water 5:95 (buffer A) and 0.1% trifluoroacetic acid in acetonitrile:water 95:5 (buffer B). A flow rate of 12.5 ml/min was applied using the following protocol: 100% buffer A for 5 min followed by a linear gradient of buffer B (0-100%) for 40 min, 100% buffer B for 5 min, another linear gradient of buffer B (100-0%) for 5 min and finally 100% buffer A for 5 min. Fractions were collected every 63 s, resulting in 57 fractions of 13 ml. One milliliter of each collected fraction was dried in a speedvac overnight. The fraction residues were dissolved in 50 µl DMSO and tested in serial dilutions, starting at 100× diluted. The sole active fraction has been analyzed using analytical chemical methods as described below.

### Identification of biologically active compounds

First, an active fraction was assessed for its purity through analytical HPLC, using a Shimadzu LC-2030 system with photodiode array (PDA) detection (190-800 nm) using a Shimadzu Shim-pack GIST C18-HP reversed-phase column (3 µm, 4.6×100 mm). Simultaneously, through PDA detection, a UV-Vis spectrum was obtained for the active compound. Second, HRMS was measured on an LCT instrument (Micromass, Manchester, UK). The sample was mixed with sodium formate, allowing the sodium formate to be used as internal calibrant, facilitating more accurate identification of the mass of the compound. The remainder of the active fraction was dried in a speedvac and dissolved in 400 ml DMSO-d6. Next, a ^1^H-nuclear magnetic resonance (NMR) spectrum was measured at 400 MHz using an Agilent-400 instrument.

### Compounds

Cercosporamide (SML0172), dorsomorphin (P5499), DMH-1 (D8946), LDN-193189 (SML0559), Alk5-inhibitor II (616452), A83-01 (SML0788) and DMSO were all purchased from Sigma-Aldrich (Zwijndrecht, The Netherlands). eFT508 (HY-100022) was obtained from Toronto Research Chemicals (Toronto, Canada). Catalog numbers are indicated in parentheses.

### *In situ* hybridization

Embryos were treated from approximately 6 hpf until 12 hpf when they were fixed in 4% paraformaldehyde overnight. *In situ* hybridization was performed using *krox20* and *myod* anti-sense RNA probes as described elsewhere (Thisse and Thisse, 2008).

### Kinase activity assay

A radiometric protein kinase assay was performed by ProQinase GmbH (Freiburg, Germany), using purified bacterially expressed human kinases and a range of concentrations of cercosporamide. IC50 values were calculated using Prism 5.04 for Windows (GraphPad, San Diego, CA, USA; www.graphpad.com). The mathematical model used was ‘Sigmoidal response (variable slope)’ with parameters ‘top’ fixed at 100% and ‘bottom’ at 0%. The fitting method used was a least-squares fit.

### Mammalian cell lines and treatment

HepG2 and HEK 293T cells were routinely grown in Dulbecco's modified Eagle medium supplemented with 10% fecal calf serum, supplemented with penicillin and streptomycin and glutamine. HepG2 and HEK 293T cell lines were obtained from American Type Culture Collection, were frequently tested for absence of mycoplasma and were authenticated using an STR profiling kit from Promega.

For BMP stimulation, the HepG2 cells (∼50% confluency) were starved on serum-free medium for 6 h. Subsequently, prior to the addition of BMP ligands, the cells were treated with compound for 30 min. Next, the cells were stimulated with BMP2 (50 ng/ml) or BMP6 (50 ng/ml) for 45 min. The cells were then washed with PBS and lysed in Laemmli sample buffer for western blot analysis.

### Immunoblot analysis

HepG2 or HEK 293T cells were lysed in Laemmli sample buffer. Proteins were separated by sodium dodecyl sulfate polyacrylamide gel electrophoresis (SDS-PAGE) and transferred onto 45-μm polyvinylidene difluoride (PVDF) membrane (IPVH00010, Merck Millipore). Membranes were blocked using 5% non-fat dry milk in Tris-buffered saline with 0.1% Tween 20 (655204, Merck Millipore) and probed with the respective primary and secondary antibodies. The signal was detected using Clarity™ Western ECL Substrate (1705060, Bio-Rad) and ChemiDoc Imaging System (17001402, Bio-Rad). The antibodies used for immunoblotting were raised against the following proteins: phospho-SMAD1/5/8 (Persson et al., 1998; 1:1000), SMAD1 (Cell Signaling Technology, #6944; 1:1000), GAPDH (Merck Millipore, #G8795; 1:5000) and hemagglutinin (HA; Roche, #12CA5; 1:1000).

### Transfections, luciferase assays and DNA constructs

For luciferase transcriptional reporter assays, HepG2 cells were seeded in 9-cm plates at ∼60% confluency and transfected with polyethyleneimine (PEI). Twenty-four hours later, the transfected cells were seeded in 24-well plates at ∼60% confluency. Another 24 h later, the cells were serum starved for 6 h. Subsequently the cells were treated with compound or DMSO for 30 min. Thereafter, the cells were stimulated with BMP2 in the presence of compounds (or DMSO) for 16 h (overnight). Subsequently, the cells were washed with PBS and lysed. Luciferase activity was measured using the luciferase reporter assay system from Promega (Leiden, The Netherlands) by a Perkin Elmer luminometer Victor^3^ 1420. Each DNA transfection mixture was equalized with empty vector when necessary and every experiment was performed in triplicate. β-galactosidase expression construct was co-transfected and β-galactosidase was measured to normalize for differences in transfection efficiency. The BRE-luc reporter construct has been reported previously (Korchynskyi and ten Dijke, 2002).

For experiments with the caALK type I receptor constructs, HEK 293T cells were seeded in 24-well plates at ∼90% confluency and transfected with the DNA expression constructs in the presence of PEI. Thirty hours after transfection, the cells were added to serum-starved medium and treated with compounds for 16 h (overnight). Subsequently, the cells were washed with PBS and lysed in Laemmli sample buffer. Expression constructs for constitutively active type I receptors (caALK1, caALK2, caALK3, caALK5, caALK6) were previously described (Dennler et al., 1998; Fujii et al., 1999).

### mRNA synthesis and micro-injection

The pCS2+ plasmid encoding caAlk2 with a glutamine to aspartic acid substitution at position 204 was kindly donated by Jeroen Bakkers (Smith et al., 2009). The DNA sequences of the inserts in plasmid constructs were verified. Both plasmids were digested with *Not*I and mRNA was generated with SP6 RNA polymerase using a mMessage mMachine kit (Ambion). The mRNA was purified through phenol/isoamylalcohol/chloroform extraction. Zebrafish embryos were injected into the yolk at the one-cell stage with ∼1 nl of either 50 ng/µl GFP mRNA or a cocktail containing 10 ng/µl caAlk2 and 50 ng/µl GFP mRNA. Subsequently, the embryos were washed with E3 medium and distributed in a 12-well plate, with 15-20 embryos per well, and incubated with either DMSO, 100 nM or 200 nM cercosporamide from 2 hpf onwards. Next, embryos were selected for fluorescence at 24 hpf and examined at 48 hpf. The phenotypes were categorized into three groups: no head; head, no eyes; head and eyes. Finally, the bar graph was generated using Prism 8.3.0 for Windows (GraphPad, San Diego, CA, USA). The presented data are a combination of two repeats of the experiment.

## Supplementary Material

Supplementary information
