# Supplementary material for: Cercosporamide inhibits bone morphogenetic protein receptor type I kinase activity in zebrafish
Source: Dis Model Mech. 2020 Sep 24;13(9):dmm045971. doi: 10.1242/dmm.045971 (PMC7522027; doi:10.1242/dmm.045971)
Supplement: Supplementary information [file dmm-13-045971-s1.pdf]

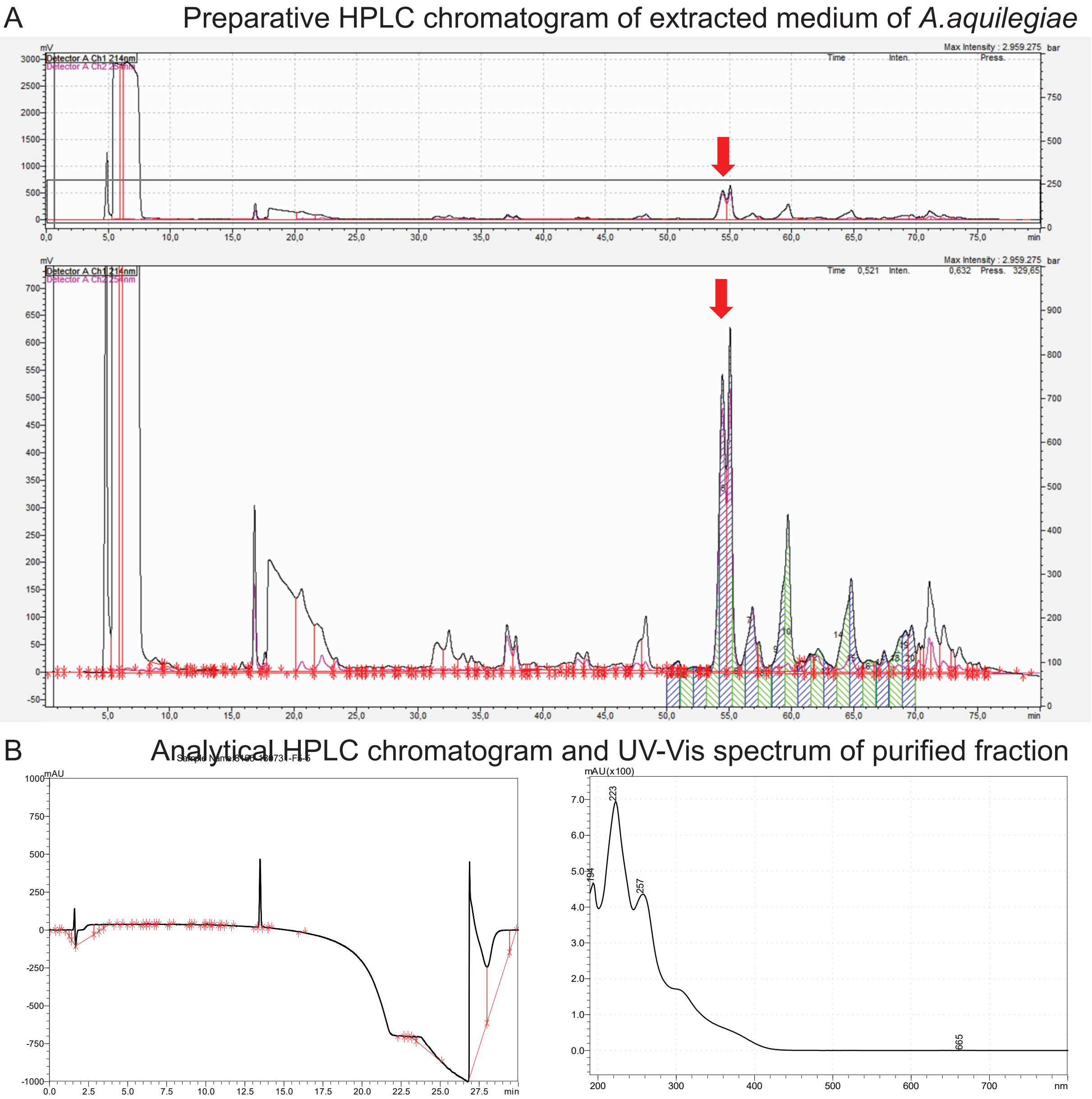

Fig. S1: (A) Purification of active fraction (red arrow) through preparative HPLC. (B) Assessment of the purity and aquirement of an UV-Vis spectrum of the active fraction through analytical HPLC with diode array detection.

Disease Models & Mechanisms • Supplementary information

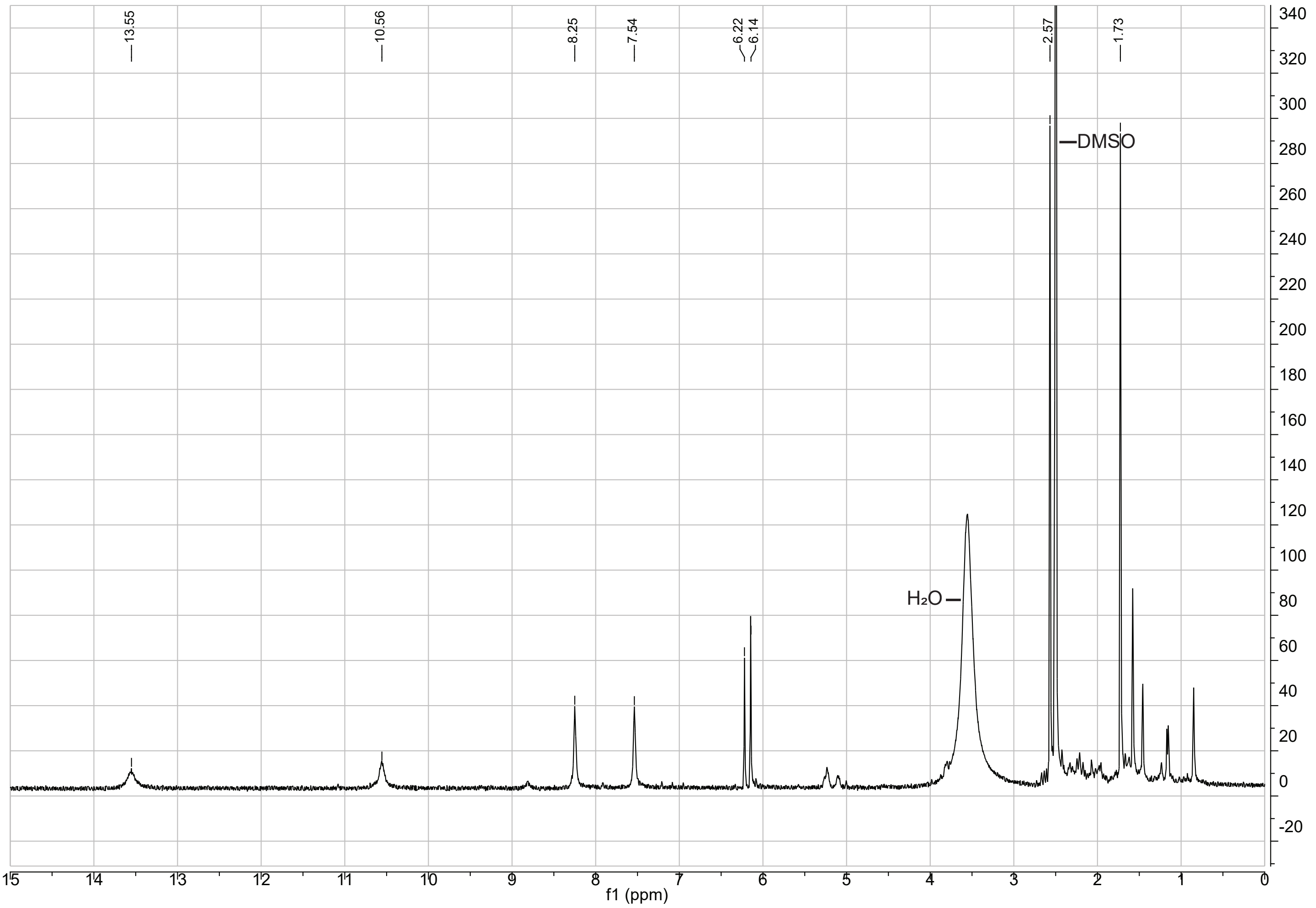

Fig. S2: ^{1}H-NMR spectrum of active preparative HPLC fraction in DMSO-d_6

Disease Models & Mechanisms • Supplementary information

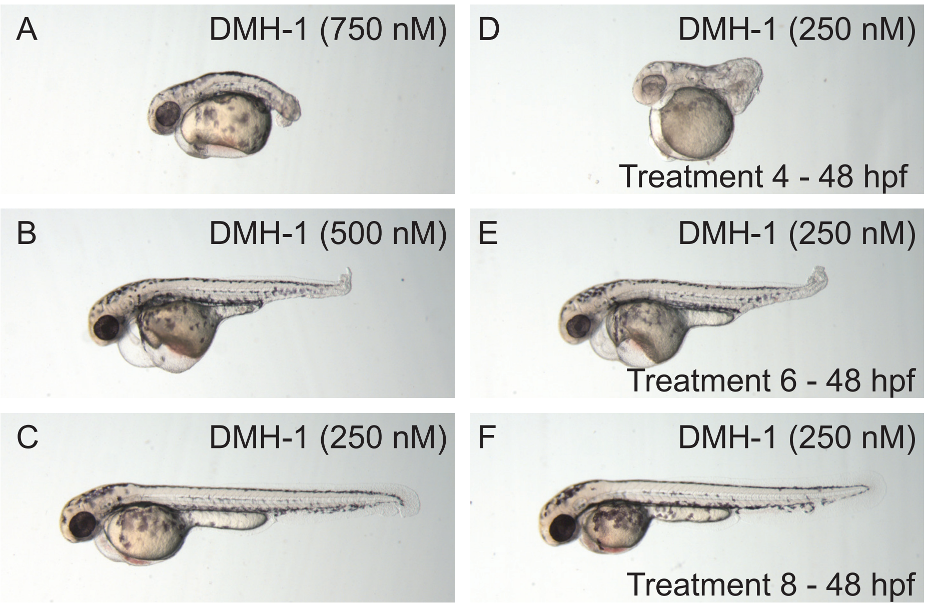

Fig. S3: Dose- and time-dependent developmental defects of DMH-1 in zebrafish embryos. (A-C) Examples of phenotypes caused by a dilution range of DMH-1 (750 nM - 250 nM). (D-F) Examples of phenotypes caused by 250 nM DMH-1 with different treatment starting times. Treatment from 2 hpf onwards is lethal. Treatment from 4 hpf is lethal in 50% of the embryos.

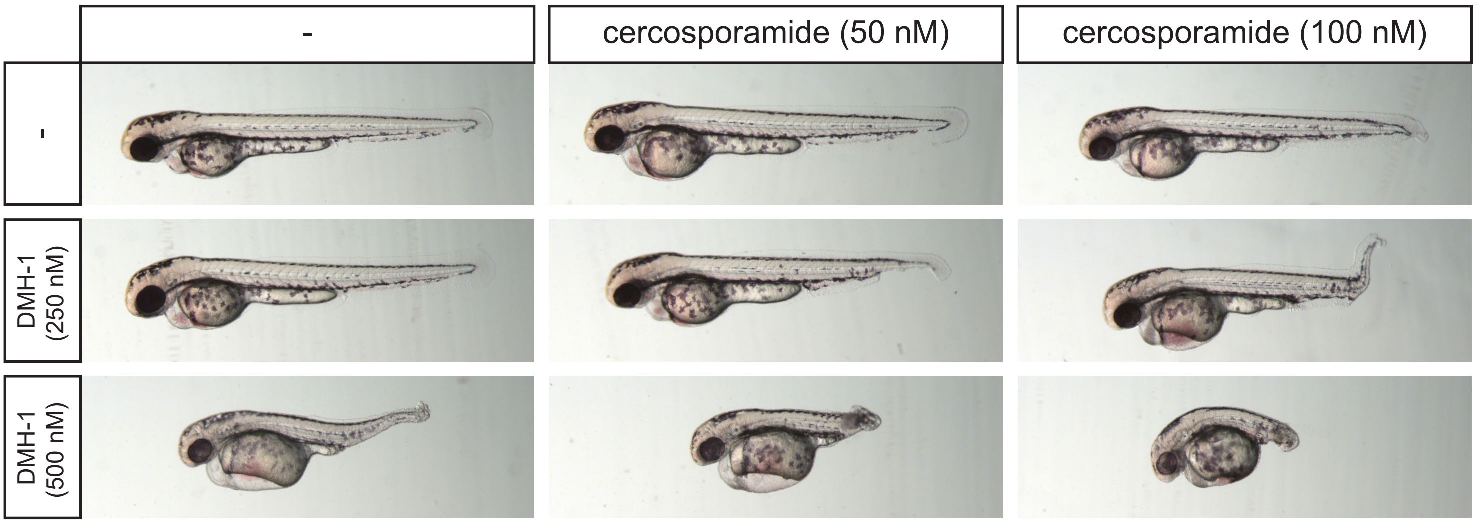

Fig. S4: Cercosporamide and known BMP inhibitors cooperate. Combination treatments of zebrafish embryos suggest that cercosporamide acts on BMP signaling pathway. Embryos were treated with cercosporamide (50 or 100 nM) or DMH-1 (250 or 500 nM).

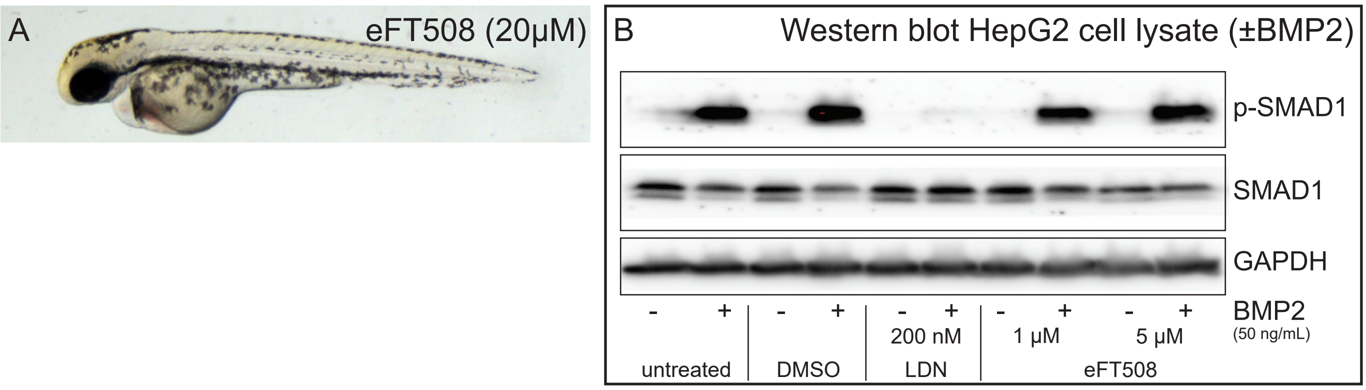

Fig. S5: Inhibition of Mnk1/Mnk2 using the potent, selective inhibitor, eFT 508 (A) did not induce developmental defects in zebrafish embryos at 20 μM, and (B) did not affect BMP2-induced SMAD 1 phosphorylation in HepG2 cells.

Disease Models & Mechanisms • Supplementary information

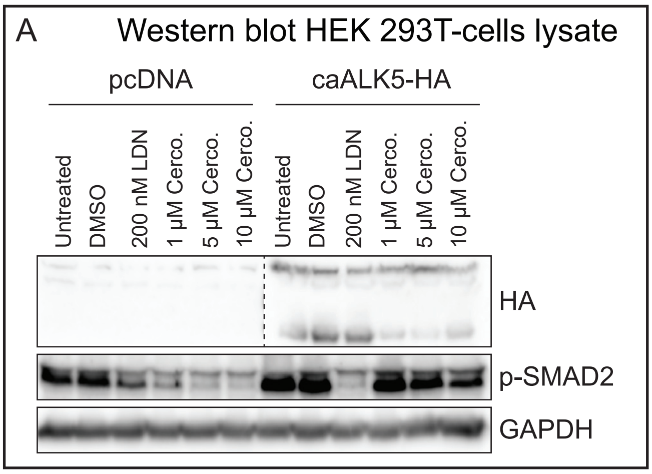

Fig. S6: caAlk5 was not inhibited by cercosporamide in transfected HEK 293T-cells.

Disease Models & Mechanisms • Supplementary information

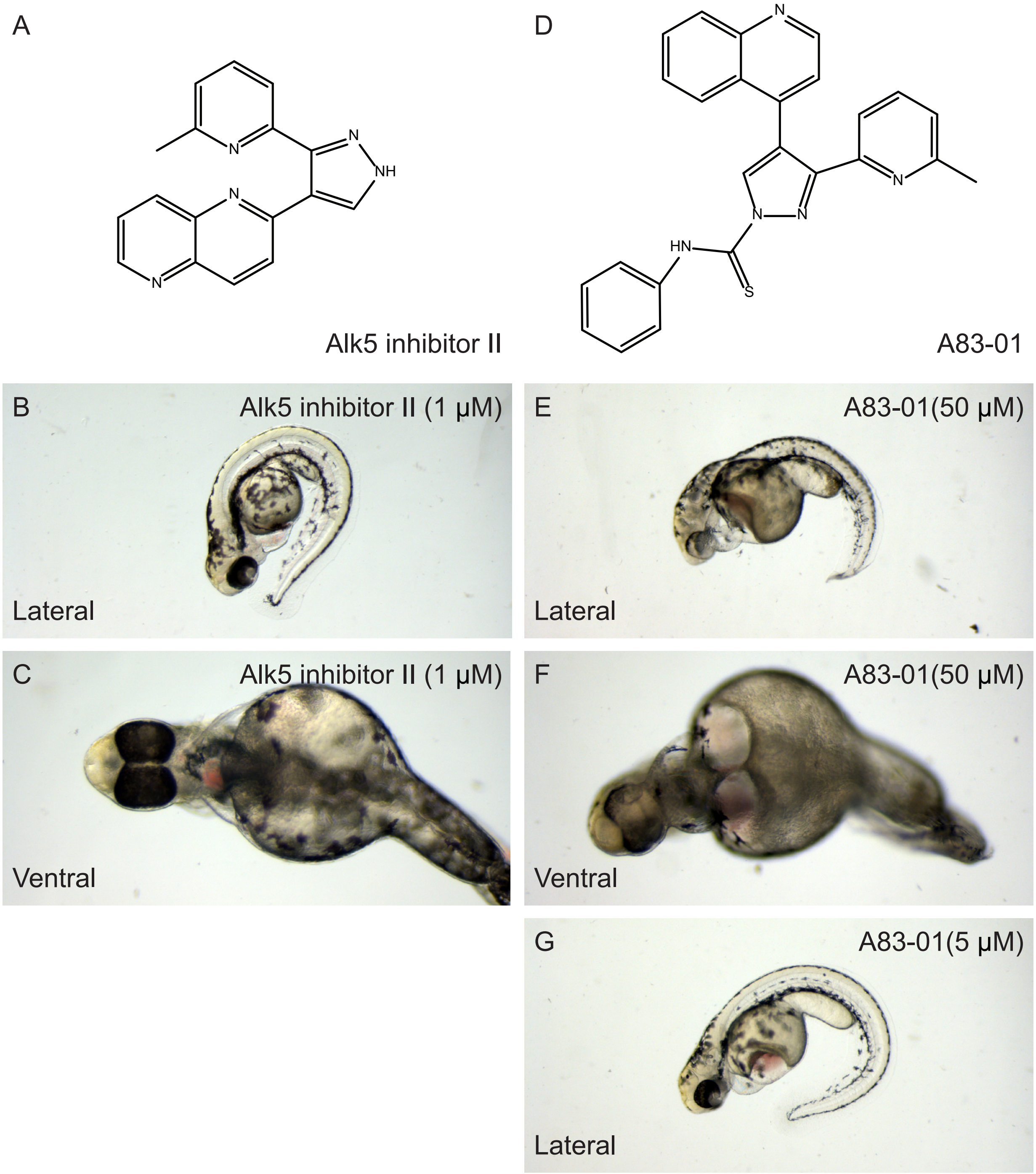

Fig. S7: Inhibition of Alk5 using two independent Alk5 inhibitors induced developmental defects that were distinct from the developmental defects induced by known BMP inhibitors and cercosporamide.
